# Action of Salicylic Acid on Plant Growth

**DOI:** 10.3389/fpls.2022.878076

**Published:** 2022-04-27

**Authors:** Aixia Li, Xue Sun, Lijing Liu

**Affiliations:** The Key Laboratory of Plant Development and Environmental Adaptation Biology, School of Life Sciences, Ministry of Education, Shandong University, Jinan, China

**Keywords:** salicylic acid, plant growth regulation, phytohormones, cell expansion, cell division, hormone crosstalk

## Abstract

The phytohormone salicylic acid (SA) not only is a well-known signal molecule mediating plant immunity, but also is involved in plant growth regulation. However, while its role in plant immunity has been well elucidated, its action on plant growth has not been clearly described to date. Recently, increasing evidence has shown that SA plays crucial roles in regulating cell division and cell expansion, the key processes that determines the final stature of plant. This review summarizes the current knowledge on the action and molecular mechanisms through which SA regulates plant growth *via* multiple pathways. It is here highlighted that SA mediates growth regulation by affecting cell division and expansion. In addition, the interactions of SA with other hormones and their role in plant growth determination were also discussed. Further understanding of the mechanism underlying SA-mediated growth will be instrumental for future crop improvement.

## Introduction

Salicylic acid (SA) is an important phytohormone that serves as a critical signal molecule mediating immunity and plant growth (Hayat et al., 2007; Vlot et al., 2009; Rivas-San Vicente and Plasencia, 2011). SA is synthesized from chorismate through two distinct pathways in plants, the isochorismate (IC) and the phenylalanine ammonia-lyase (PAL) pathways (Serino et al., 1995; Zhang and Li, 2019). In *Arabidopsis*, the majority (>90%) of SA is produced *via* the IC pathway, which contains two isochorismate synthase (ICS) enzymes (ICS1, also known as salicylic acid induction deficient 2, SID2, and ICS2) and two other enzymes, namely, *avrPphB* susceptible 3 and enhanced *pseudomonas* susceptibility 1 (Wildermuth et al., 2001; Zhang and Li, 2019). In contrast, in rice, the PAL pathway, which contains nine PAL enzymes and an abnormal inflorescence meristem 1 (AIM1) enzyme, may constitute the predominant pathway for SA synthesis (Silverman et al., 1995; Tonnessen et al., 2015). SA is perceived by the nonexpressor of pathogenesis-related gene 1 (NPR1) and its paralogues NPR3 and NPR4. It subsequently stimulates the downstream SA responsive genes and induces plant immune response (Fu et al., 2012; Wu et al., 2012; Ding et al., 2018). In addition to the canonical SA receptor NPRs (NPR1/NPR3/NPR4), there are many other SA-binding proteins (SABPs), such as SABP1 (Catalase), SABP2 (MeSA Esterase), and SABP3 (β carbonic anhydrase), which may act as potential SA receptors in plants and may be involved in SA signaling (Pokotylo et al., 2019). SA could be modified through glycosylation, methylation, and amino acid (AA) conjugation to render it inactive or fine-tune its accumulation, function, and/or mobility (Dempsey and Klessig, 2017; Ding and Ding, 2020), and thus affecting the regulation of SA on plant immunity and growth.

The final stature of plant growth is manifested by cell number and cell size, which are mainly controlled by the cell division and expansion processes, and these are determined by both genetic constraints and environmental signals (Mizukami, 2001; Weiss et al., 2005; Tsukaya, 2019). In the past two decades, increasing evidence has shown that SA plays essential roles in regulating plant growth by affecting cell division and cell expansion (Vanacker et al., 2001; Scott et al., 2004; Miura et al., 2010; Fujikura et al., 2020). However, the action of SA in the regulation of plant growth has not been comprehensively described yet in most reviews on this topic, compared to the role of other plant hormones, such as auxin and gibberellin (GA; Santner et al., 2009; Wolters and Jürgens, 2009; Depuydt and Hardtke, 2011; Vanstraelen and Benková, 2012; Ali et al., 2020). This mini-review mainly describes the versatile action and molecular mechanisms of SA regulating plant and organ growth. This knowledge of the SA-mediated growth regulation will contribute to the future crop improvement.

## Effect of SA on Plant Growth

The altered endogenous SA levels in plants can result in abnormal growth phenotypes (Rivas-San Vicente and Plasencia, 2011; Pokotylo et al., 2021). A high level of endogenous SA induces a stunted stature. Many *Arabidopsis* mutants with overaccumulating SA, such as accelerated cell death 6 (*acd6*), constitutive expresser of PR gene 5 *(cpr5)*, and the *SAP* and MIZ domain protein gene 1 (*siz1*), present the dwarf plant phenotype with a shorter stem, smaller leaves, and/or floral organs compared to the wild type (WT; Bowling et al., 1997; Rate et al., 1999; Miura et al., 2010). On the contrary, the *Arabidopsis* SA-deficient mutant *sid2* and SA-depleted *NahG* transgenic line show increased leaf biomass compared to WT (Scott et al., 2004; Abreu and Munné-Bosch, 2009). It is worth noting that SA may have divergent effects in different plant species or organs. For example, the *aim1* rice mutant with reduced endogenous SA levels has shorter seedling and adventitious roots compared to the wild type (Xu et al., 2017).

The effect of exogenous SA on growth depends on its concentration and on the plant species. Different SA concentrations have either promoting or inhibiting effects on plant and organ growth in different plant species (Table 1). Studies have shown that a 0.01 mm SA treatment increased rosette diameter and the number of leaves and flower buds in African violet (Jabbarzadeh et al., 2009), while 0.05 mm SA stimulated the growth of wheat seedlings and the formation of larger ears (Shakirova et al., 2003). In finger millet, 0.1 mm SA stimulated flowering and grain set (Appu and Muthukrishnan, 2014) and, in strawberry, 0.25 mm SA caused a significant increase in leaf area and weight of primary fruits (Kazemi, 2013). In addition, 0.5 mm SA enhanced dry weight of root, shoot and nodule, and the number of flower and pods in chickpea (Kaur et al., 2022), and it also significantly increased photosynthesis and growth in wheat and mungbean (Khan et al., 2013, 2014). However, in *Arabidopsis*, exogenous SA (0.02–0.03 mm) reduced pollen tube length by about 25% (Rong et al., 2016), and SA treatments (0.1 and 1 mm) also decreased trichome density and number (Traw and Bergelson, 2003). In tobacco, 0.1 mm SA reduced shoot growth and leaf epidermal cell size (Dat et al., 2000). Pancheya et al. (1996) observed that SA concentrations between 0.1 and 1 mm inhibited the growth of leaves and roots of barley seedlings in a dosage-dependent manner. Generally, for a plant species, lower concentrations of exogenous SA seem to have a growth-promoting effect while higher ones may negatively regulate growth (Table 1). For example, 0.05 mm SA significantly stimulated the growth of rosette leaves and roots of chamomilla by 32 and 65%, respectively, while 0.25 mm SA decreased it by 40 and 43%, respectively (Kovácik et al., 2009). In *Arabidopsis*, low-concentration SA (<0.05 mm) promoted adventitious roots, whereas high-concentration SA (>0.05 mm) inhibited all growth processes in the root (Pasternak et al., 2019). The discrepant performances of different concentration SA on growth were also reported in wheat and pepper (Hayat et al., 2005; Canakci, 2011). It should be mentioned that the threshold SA concentration between growth promotion and growth inhibition may vary with plant species. These contrasting effects due to different concentrations of exogenous SA indicate that this compound has a complex role in plant growth.

**Table 1:** Effect of exogenous SA on growth in different plant species.

Plant species	SA conc.	Effect	References
African violet	0.01 mm	Increased rosette diameter and the number of leaves and flower buds.	Jabbarzadeh et al., 2009
Finger millet	0.1 mm	Stimulated flowering and grain set.	Appu and Muthukrishnan, 2014
Strawberry	0.25 mm	Increased leaf area and weight of primary fruits.	Kazemi, 2013
Chickpea	0.5 mm	Enhanced dry weight of root, shoot and nodule, and the number of flower and pods.	Kaur et al., 2022
Mungbean	0.5 mm	Promoted photosynthesis and growth.	Khan et al., 2014
Tobacco	0.1 mm	Reduced shoot growth and leaf epidermal cell size.	Dat et al., 2000
Barley	0.1–1 mm	Inhibited the growth of leaves and roots of seedlings.	Pancheya et al., 1996
Wheat	0.05 mm	Stimulated the growth of young seedlings and the occurrence of larger ears.	Shakirova et al., 2003
	0.5 mm	Promoted photosynthesis.	Khan et al., 2013
	0.01 and 1 mm	Low-concentration SA increased fresh and dry plant weight, whereas high-concentration SA decreased it.	Hayat et al., 2005
Chamomilla	0.05 and 0.25 mm	Low-concentration SA stimulated the growth of rosette leaves and roots by 32 and 65%, respectively, whereas high-concentration SA decreased it by 40 and 43%, respectively.	Kovácik et al., 2009
Arabidopsis	0.02–0.03 mm	Reduced pollen tube length by about 25%.	Rong et al., 2016
	<0.05 mm, >0.05 mm	Lower concentrations SA promoted adventitious roots, whereas higher concentrations SA inhibited all growth processes in the root.	Pasternak et al., 2019
	0.1 and 1 mm	Decreased trichome density and number.	Traw and Bergelson, 2003
Pepper	1.5 and 10 mm	Low-concentration SA has a stimulating effect on seedling length and fresh weight, whereas high-concentration SA has an inhibiting effect on it.	Canakci, 2011

## SA-Induced Regulation of Cell Division and Expansion

SA can regulate plant growth by modulating cell division and expansion, either in a negative or positive way. In *Arabidopsis* leaves, some evidence has shown that SA has a negative effect on the two cellular processes. For instance, SA-deficient *NahG* transgenic plants displayed a higher growth rate compared to WT and they presented a 1.7-fold increase in leaf rosette biomass at the early stage of reproduction (Abreu and Munné-Bosch, 2009). This increased growth effect on *NahG* transgenic plants was more obvious at low temperatures, and it resulted from enhanced cell expansion of rosette leaves (Scott et al., 2004; Xia et al., 2009). Further investigations have demonstrated that, compared with WT, *NahG* transgenic plants at 4°C presented an elevated expression of the cell cycle G1/S transition regulator cyclin D 3 (CYCD3) and enhanced endoreduplication levels, which led to larger cells (Xia et al., 2009). This evidence indicated that SA suppresses cell expansion by regulating the expression of the cyclin genes. In addition, the SA-accumulating mutant *siz1* showed a dwarf phenotype characterized by a decreased leaf cell volume and number. The cell division and expansion defects caused by *siz1* can be suppressed through the overexpression of *NahG* (Miura et al., 2010), further demonstrating that SA inhibits these two cellular processes. However, SA accumulation in a different context may exhibit a discrepant action on cell growth control. Vanacker et al. (2001) found that SA activates cell division and expansion in *acd6-1* leaves with a very high SA level. Additionally, the positive effect of SA on cell division was also found in the roots. For example, the *aim1* rice mutant, whose SA biosynthesis was deficient, showed a reduced root meristem activity and a significantly lower expression of the cell cycle G2/M phase transition regulator cyclin B1;1 (CYCB1;1) compared to WT, indicating that SA has a positive role in root cell division (Xu et al., 2017). Moreover, the SA-overaccumulating *Arabidopsis* mutant known as *constitutively activated cell death 1* (*cad1*) increased cell division in the quiescent center (QC) in the root apical meristem, which was observed in the SA-treated WT (Wang et al., 2021). In summary, the SA-induced regulation of cell division and expansion is complicated and may depend on plant organs and the context in which signaling occurs.

## SA-Induced Regulation of Plant Growth *via* Multiple Signaling Pathways

SA signaling during plant immune responses has been well explored, and NPR1 was identified as a key component of this process. NPR1 is also important for SA-mediated growth regulation through the control of cell division and expansion (Vanacker et al., 2001; Fujikura et al., 2020; Wang et al., 2021). The *Arabidopsis npr1-1* mutant leaves had fewer cells and higher DNA content, indicating that NPR1 promotes cell division and represses endoreduplication in leaves (Vanacker et al., 2001). Fujikura et al. (2020) found that the *xs2* mutant accumulated high SA contents and impaired cell expansion, producing smaller cells compared to those observed in WT. Interestingly, the significant defect in cell size observed in the *xs2* mutant was restored in the *xs2 npr1* double mutant. These results indicate that the suppression of cell expansion in *xs2* was mediated through an NPR1-dependent signaling pathway. Additionally, the SA-overaccumulating *Arabidopsis* mutant *cad1* increased cell division in the QC, which was rescued through the mutation of *SID2* or *NPR1*, indicating that the QC cell division in the *cad1* mutant is promoted in an NPR1-dependent SA signaling pathway (Wang et al., 2021). Further investigation found that SA accumulation in the *cad1* mutant promotes QC cell division through the accumulation of reactive oxygen species and downregulation of the transcription factor genes *Plethora 1* (*PLT1*), *PLT2*, and *WUSCHEL-related homeobox5* (*WOX5*) involved in QC maintenance (Wang et al., 2021). NPR1 may also have a negative effect on cell division in some specific developmental contexts, such as the SA-Ethylene (ET)-mediated apical hook development (Raz and Koornneef, 2001; Huang et al., 2020). The above examples support the hypothesis that SA could regulate cell division, cell expansion, and then plant growth in an NPR1-dependent manner.

Besides the NPR family proteins, other SABPs also play essential roles in SA-mediated plant growth regulation (Pokotylo et al., 2019). Tan et al. (2020) found that SA directly binds to A subunits of protein phosphatase 2A (PP2A) to suppress the dephosphorylation of PIN-formed (PIN) auxin efflux carriers and inhibit root development (including root elongation, gravity response, and lateral root formation) in an NPR-independent manner. Additionally, the inhibiting effect of SA on pollen tube growth in *Arabidopsis* is also independent of NPRs because the *npr1*, *npr3*, *npr4*, and *npr3 npr4* mutants exhibited responses that were identical to that of 20 mm SA in WT (Rong et al., 2016). Further investigations revealed that SA and methylated SA (MeSA) antagonistically regulate pollen tube growth by affecting clathrin-mediated endocytosis (CME) and the apical activation of a key pollen tip growth regulator, known as Rho-type GTPase 1 (ROP1), with SA and MeSA having inhibitory and stimulatory effects, respectively. Interestingly, the methylesterase and methyltransferase enzymes, which catalyze the interconversion between SA and MeSA, are both localized on the tip of pollen tubes, indicating that the tip-localized production of the two compounds plays an important role in the regulation of polar cell growth (Rong et al., 2016). However, it remains to be determined which SABP is responsible for the SA/MeSA-mediated regulation of pollen tube growth.

Other SA pathways regulating plant growth have also been reported. The *Arabidopsis cpr5* containing an elevated SA level was shown to reduce primary root length and lateral root number, and these could be restored in the *cpr5/sid2* double mutant (Bowling et al., 1997). Additional research demonstrated that CPR5 can repress the accumulation of SA and favor growth through the inhibition of the SA-dependent inositol-requiring protein-1 (IRE1)-basic leucine zipper 60 (bZIP60) arm-induced plant unfolded protein response (UPR), which antagonizes organ growth (Meng et al., 2017), indicating that CPR5 regulates root growth through the SA-dependent IRE1-bZIP60 signaling pathway. Moreover, SA also regulates plant growth by affecting the expression of genes involved in cell expansion. In the *siz1* mutant mentioned above, SA accumulation negatively regulates the expression of the xyloglucan endotransglycosylase/hydrolase (XTH) genes, *XTH8* and *XTH31*, which facilitate cell wall loosening and cell expansion in leaves (Miura et al., 2010). Nevertheless, the SABPs involved in these pathways have not been explored yet.

The above-mentioned studies show that the action of SA on organ and plant growth is mediated by different receptors, and it occurs through multiple pathways, supporting the hypothesis that SA plays a complex role in plant growth regulation.

## Role of SA Crosstalk With Other Hormones in the Regulation of Plant Growth

SA also interacts with other hormones involved in the regulation of cell division and expansion, such as auxin, GA, and ethylene (ET), to modulate plant and organ growth (Ari et al., 2020; Emamverdian et al., 2020; Mazzoni-Putman et al., 2021; Pokotylo et al., 2021).

Auxin is a major growth hormone that controls these cellular processes, especially in roots (Perrot-Rechenmann, 2010; Barbeza et al., 2017; Huang et al., 2019; Seo et al., 2021), and SA can influence root development by affecting the accumulation and transport of auxin. Pasternak et al. (2019) proved that exogenous SA affects the root tip meristem in *Arabidopsis* in a concentration-dependent manner. A low level of SA (below 0.05 mM) induces auxin accumulation by activating the expression of the auxin biosynthetic enzyme TRP aminotransferase of *Arabidopsis* 1 (TAA1) and the auxin efflux protein PIN1, and by repressing PIN2 and PIN7. Then, it increases the number of periclinal and tangential cells in the roots’ outer layers through a cyclin D6;1 (CYCD6;1)-dependent mechanism. In contrast, a high dosage of SA (above 0.05 mM) induces auxin depletion in the root meristem by repressing the expression of PIN1, PIN2, and PIN7, and then inhibiting the cell cycle and growth processes in the roots (Pasternak et al., 2019). SA can also lead to the hyperphosphorylation of PIN2 by directly binding to the PP2A to repress its dephosphorylation activity toward PIN2, thereby reducing PIN activity, which results in a decrease in auxin export and attenuation of root growth (Tan et al., 2020). Furthermore, SA can induce PIN2 hyperclustering through a remorin (REM)-dependent lipid nanodomain organization to hamper auxin accumulation and impair the auxin-mediated root gravitropic response (Ke et al., 2021).

GA promotes plant growth by increasing cell division and expansion (Achard et al., 2009; Sprangers et al., 2020; Vercruysse et al., 2020). The application of exogenous GA increases SA biosynthesis in wild *Arabidopsis* plants or promotes seed germination and seedling growth in the *sid2* under adverse conditions, suggesting a synergistic interplay between SA and GA (Alonso-Ramírez et al., 2009). However, SA can also antagonize GA in growth regulation. In barley, SA treatments were shown to inhibit the GA-induced alpha-amylase expression in the aleurone layers and to influence seed germination and subsequent growth (Xie et al., 2007). In *Arabidopsis*, GA increases trichome number, while this effect is suppressed by SA (Traw and Bergelson, 2003). Moreover, the hydroxycinnamoyl CoA: shikimate hydroxycinnamoyl transferase *(HCT)-RNAi* lines of *Arabidopsis* characterized by overaccumulating SA reduced the expression of GA marker genes and caused severe dwarfism in the plant. The defects of *HCT RNAi* lines in GA signaling and growth could be restored by mutating *SID2* or overexpressing *NahG* (Gallego-Giraldo et al., 2011). It is therefore inferred that SA negatively regulates cell division, cell expansion, and overall plant growth, by antagonizing GA.

ET is also an endogenous regulator of cell division and expansion in vegetative growth (Dubois et al., 2018). It participates to the stimulation of cell division during the early development of apical hooks (Raz and Koornneef, 2001). SA can inhibit ET biogenesis and signaling (Leslie and Romani, 1988; Huang et al., 2020) and, in *Arabidopsis*, it was shown to reduce the apical hook angle of etiolated seedlings by antagonizing ET signaling. The SA-activated NPR1 directly interacts with ethylene insensitive 3 (EIN3), the core transcription factor of ET, and disrupts the binding of EIN3 to its target gene promoters, such as the promoter of HOOKLESS 1 which is essential for apical hook development, thus reducing the hook angle (Huang et al., 2020). Therefore, SA may have a negative effect on cell division by antagonizing ET.

## Conclusion and Perspectives

**Figure 1:**
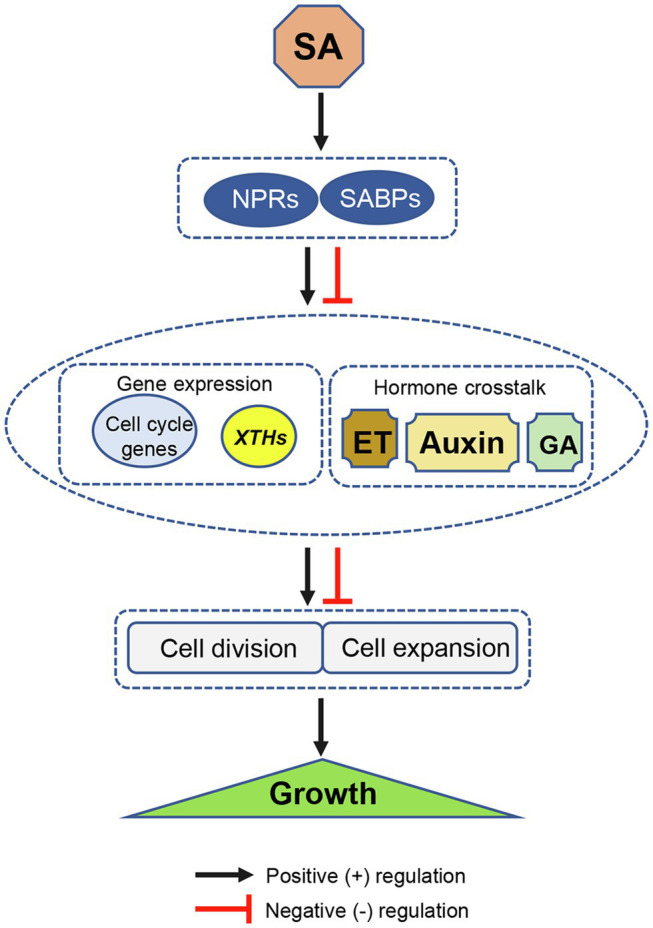
Schematic diagram illustrating a possible mode of SA-induced regulation of cell division and expansion with effects on plant growth. SA positively or negatively modulates cell division and expansion by regulating the transcription of key genes (such as cell cycle related- and cell wall loosening genes *XTHs*), and through hormone crosstalk in NPR1-dependent or other SABP-dependent manners. XTHs, xyloglucan endotransglycosylase/hydrolase genes; NPR1, nonexpressor of pathogenesis-related gene 1; and SABP, SA-binding protein. Black arrows and red T bars indicate positive (+) and negative (−) regulation, respectively.

Over past decades, numerous studies have focused on the role and mode of action of SA in plant immunity (Zhang and Li, 2019; Ding and Ding, 2020). A few studies have identified some molecular connections between SA and cell division, cell expansion, and growth. The uncovered pathways shown in this mini-review, such as SA-dependent plant UPR pathways, SA-regulated expression of cell cycle genes, and SA-auxin crosstalk, were species-, organ-, or context-dependent. The universal and key connections of SA modulating cell division and expansion are still vague and remain to be identified. Based on the current available evidence, a simple model describing SA-induced regulation of cell division and expansion with effects on plant growth was here proposed (Figure 1). It suggests that NPRs or other SABPs bind to SA to modulate the transcription of key genes (such as those associated with the cell cycle and cell wall loosening) or crosstalk with other hormones (such as auxin, GA, and ET) in either positive or negative way, and then regulate cell division or expansion, ultimately modifying plant growth.

It must be noted the higher accumulation of endogenous SA enhances plant immunity but generally suppresses growth (Van Butselaar and Van den Ackerveken, 2020). Nevertheless, SA separately regulates plant growth and immunity through different receptors or pathways in some cases. Therefore, it seems feasible to disrupt the growth-immunity tradeoff to promote defense on the premise of maintaining plant growth, or simultaneously enhance plant growth and defense *via* targeted gene editing. In summary, this mini-review on the action and mechanism of SA-induced growth regulation will provide helpful information for future crop improvement.

## Author Contributions

LL and AL conceived this review. AL, LL, and XS wrote the manuscript and approved the final version. All authors contributed to the article and approved the submitted version.

## Funding

This work is supported by Natural Science Foundation of China (32101685 to AL), the Fundamental Research Funds of Shandong University (61200079614090 to AL), and the Natural Science Foundation of Shandong province (ZR2020MC026 to LL).

## Conflict of Interest

The authors declare that the research was conducted in the absence of any commercial or financial relationships that could be construed as a potential conflict of interest.

## Publisher’s Note

All claims expressed in this article are solely those of the authors and do not necessarily represent those of their affiliated organizations, or those of the publisher, the editors and the reviewers. Any product that may be evaluated in this article, or claim that may be made by its manufacturer, is not guaranteed or endorsed by the publisher.

## References

[ref1] Abreu M. E. Munné-Bosch S. (2009). Salicylic acid deficiency in *NahG* transgenic lines and *sid2* mutants increases seed yield in the annual plant *Arabidopsis thaliana*. J. Exp. Bot. 60, 1261–1271. doi: 10.1093/jxb/ern363, PMID: 19188277 PMC2657544

[ref2] Achard P. Gusti A. Cheminant S. Alioua M. Dhondt S. Coppens F.. (2009). Gibberellin signaling controls cell proliferation rate in *Arabidopsis*. Curr. Biol. 19, 1188–1193. doi: 10.1016/j.cub.2009.05.059, PMID: 19576768

[ref3] Ali S. Khan N. Xie L. (2020). Molecular and hormonal regulation of leaf morphogenesis in *Arabidopsis*. Int. J. Mol. Sci. 21:5132. doi: 10.3390/ijms21145132, PMID: 32698541 PMC7404056

[ref4] Alonso-Ramírez A. Rodríguez D. Reyes D. Jiménez J. A. Nicolás G. López-Climent M.. (2009). Cross-talk between gibberellins and salicylic acid in early stress responses in *Arabidopsis thaliana* seeds. Plant Signal. Behav. 4, 750–751. doi: 10.4161/psb.4.8.9175, PMID: 19820299 PMC2801389

[ref5] Appu M. Muthukrishnan S. (2014). Foliar application of salicylic acid stimulates flowering and induce defense related proteins in finger millet plants. J. Sci. Food Agric. 2, 14–18. doi: 10.13189/ujps.2014.020102

[ref6] Ari Y. Sam F. Siddiqui H. Bajguz A. Hayat S. (2020). Salicylic acid in relation to other phytohormones in plant: A study towards physiology and signal transduction under challenging environment. Environ. Exp. Bot. 175:104040. doi: 10.1016/j.envexpbot.2020.104040

[ref7] Barbeza E. Dünserb K. Gaidoraa A. Lendla T. Buscha W. (2017). Auxin steers root cell expansion via apoplastic pH regulation in *Arabidopsis thaliana*. P. Natl. Acad. Sci. U. S. A. 114, E4884–E4893. doi: 10.1073/pnas.1613499114, PMID: 28559333 PMC5474774

[ref8] Bowling S. A. Clarke J. D. Liu Y. Klessig D. F. Dong X. (1997). The *cpr5* mutant of *Arabidopsis* expresses both NPR1-dependent and NPR1-independent resistance. Plant Cell 9, 1573–1584. doi: 10.1105/tpc.9.9.1573, PMID: 9338960 PMC157034

[ref9] Canakci S. (2011). Effect of salicylic acid on growth, biochemical constituents in pepper (*Capsicum annuum L*.) seedlings. Pak. J. Biol. Sci. 14, 300–304. doi: 10.3923/pjbs.2011.300.304, PMID: 21870633

[ref10] Dat J. F. Lopez-Delgado H. Foyer C. H. Scotth I. M. (2000). Effects of salicylic acid on oxidative stress and thermotolerance in tobacco. J. Plant Physiol. 156, 659–665. doi: 10.1016/S0176-1617(00)80228-X

[ref11] Dempsey D. A. Klessig D. F. (2017). How does the multifaceted plant hormone salicylic acid combat disease in plants and are similar mechanisms utilized in humans? BMC Biol. 15:23. doi: 10.1186/s12915-017-0364-8, PMID: 28335774 PMC5364617

[ref12] Depuydt S. Hardtke C. S. (2011). Hormone signalling crosstalk in plant growth regulation. Curr. Biol. 21, R365–R373. doi: 10.1016/j.cub.2011.03.013 21549959

[ref13] Ding P. T. Ding Y. L. (2020). Stories of salicylic acid: a plant defense hormone. Trends Plant Sci. 25, 549–565. doi: 10.1016/j.tplants.2020.01.004, PMID: 32407695

[ref14] Ding Y. L. Sun T. J. Ao K. Peng Y. J. Zhang Y. X. Li X.. (2018). Opposite roles of salicylic acid receptors NPR1 and NPR3/NPR4 in transcriptional regulation of plant immunity. Cell 173, 1454–1467.e15. doi: 10.1016/j.cell.2018.03.044, PMID: 29656896

[ref15] Dubois M. Van den Broeck L. Inzél D. (2018). The pivotal role of ethylene in plant growth. Trends Plant Sci. 23, 311–323. doi: 10.1016/j.tplants.2018.01.003, PMID: 29428350 PMC5890734

[ref16] Emamverdian A. Ding Y. Mokhberdoran F. (2020). The role of salicylic acid and gibberellin signaling in plant responses to abiotic stress with an emphasis on heavy metals. Plant Signal. Behav. 15:1777372. doi: 10.1080/15592324.2020.1777372, PMID: 32508222 PMC8570706

[ref17] Fu Z. Q. Yan S. Saleh A. Wang W. Ruble J. Oka N.. (2012). NPR3 and NPR4 are receptors for the immune signal salicylic acid in plants. Nature 486, 228–232. doi: 10.1038/nature11162, PMID: 22699612 PMC3376392

[ref18] Fujikura U. Kazune E. Horiguchi G. Seo M. Yuri K. Yuji K.. (2020). Suppression of class I compensated cell enlargement by *xs2* mutation is mediated by salicylic acid signaling. PLoS Genet. 16:e1008873. doi: 10.1371/journal.pgen.1008873, PMID: 32584819 PMC7343186

[ref19] Gallego-Giraldo L. Escamilla-Trevino L. Jackson L. A. Dixon R. A. (2011). Salicylic acid mediates the reduced growth of lignin down-regulated plants. P. Natl. Acad. Sci. U. S. A. 108, 20814–20819. doi: 10.1073/pnas.1117873108, PMID: 22123972 PMC3251142

[ref20] Hayat S. Ali B. Ahmad A. (2007). “Salicylic acid: biosynthesis, metabolism and physiological role in plants,” in SALICYLIC ACID: A Plant Hormone. eds. Hayat S. Ahmad A. (Netherlands: Springer), 1–14.

[ref21] Hayat S. Fariduddin Q. Ali B. Ahmad A. (2005). Effect of salicylic acid on growth and enzyme activities of wheat seedlings. Acta Agron. Hung. 53, 433–437. doi: 10.1556/AAgr.53.2005.4.9

[ref22] Huang P. X. Dong Z. Guo P. X. Zhang X. Qiu Y. P. Li B. S.. (2020). Salicylic acid suppresses apical hook formation via NPR1-mediated repression of EIN3 and EIL1 in *Arabidopsis*. Plant Cell 32, 612–629. doi: 10.1105/tpc.19.00658, PMID: 31888966 PMC7054027

[ref23] Huang R. F. Zheng R. He J. Zhou Z. M. Wang J. C. Xiong Y.. (2019). Noncanonical auxin signaling regulates cell division pattern during lateral root development. P. Natl. Acad. Sci. U. S. A. 116, 21285–21290. doi: 10.1073/pnas.1910916116, PMID: 31570617 PMC6800413

[ref24] Jabbarzadeh Z. Khosh-Khui M. Salehi H. (2009). The effect of foliar-applied salicylic acid on flowering of African violet. Aust. J. Basic Appl. Sci. 3, 4693–4696.

[ref25] Kaur H. Hussain S. J. Kaur G. Poor P. Alamri S. Siddiqui M. H.. (2022). Salicylic acid improves nitrogen fixation, growth, yield and antioxidant defence mechanisms in chickpea genotypes under salt stress. J. Plant Growth Regul. doi: 10.1007/s00344-022-10592-7

[ref26] Kazemi M. (2013). Foliar application of salicylic acid and calcium on yield, yield component and chemical properties of strawberry. Bull. Env. Pharmacol. Life Sci. 2, 19–23.

[ref27] Ke M. Y. Ma Z. M. Wang D. Y. Sun Y. B. Wen C. J. Huang D. Q.. (2021). Salicylic acid regulates PIN2 auxin transporter hyperclustering and root gravitropic growth via Remorin-dependent lipid nanodomain organisation in *Arabidopsis thaliana*. New Phytol. 229, 963–978. doi: 10.1111/nph.16915, PMID: 32901934 PMC7821329

[ref28] Khan M. I. R. Asgher M. Khan N. A. (2014). Alleviation of salt-induced photosynthesis and growth inhibition by salicylic acid involves glycinebetaine and ethylene in mungbean (*Vigna radiata* L.). Plant Physiol. Biochem. 80, 67–74. doi: 10.1016/j.plaphy.2014.03.026, PMID: 24727790

[ref29] Khan M. R. I. Iqbal N. Masood A. Per T. S. Khan N. A. (2013). Salicylic acid alleviates adverse effects of heat stress on photosynthesis through changes in proline production and ethylene formation. Plant Signal. Behav. 8:e26374. doi: 10.4161/psb.26374, PMID: 24022274 PMC4091357

[ref30] Kovácik J. Grúz J. Backor M. Strnad M. Repcák M. (2009). Salicylic acid-induced changes to growth and phenolic metabolism in Matricaria chamomilla plants. Plant Cell Rep. 28, 135–143. doi: 10.1007/s00299-008-0627-5, PMID: 18972114

[ref31] Leslie C. A. Romani R. J. (1988). Inhibition of ethylene biosynthesis by salicylic acid. Plant Physiol. 88, 833–837. doi: 10.1104/pp.88.3.833, PMID: 16666393 PMC1055670

[ref32] Mazzoni-Putman S. M. Brumos J. Zhao C. Alonso J. M. Stepanova N. A. (2021). Auxin interactions with other hormones in plant development. Cold Spring Harb. Perspect. Biol. 13:a039990. doi: 10.1101/cshperspect.a039990, PMID: 33903155 PMC8485746

[ref33] Meng Z. Ruberti C. Gong Z. Brandizzi F. (2017). CPR5 modulates salicylic acid and the unfolded protein response to manage tradeoffs between plant growth and stress responses. Plant J. 89, 486–501. doi: 10.1111/tpj.13397, PMID: 27747970 PMC5340296

[ref34] Miura K. Lee J. Miura T. Hasegawa P. M. (2010). SIZ1 controls cell growth and plant development in *Arabidopsis* through salicylic acid. Plant Cell Physiol. 51, 103–113. doi: 10.1093/pcp/pcp171, PMID: 20007967

[ref35] Mizukami Y. (2001). A matter of size: developmental control of organ size in plants. Curr. Opin. Plant Biol. 4, 533–539. doi: 10.1016/S1369-5266(00)00212-0, PMID: 11641070

[ref36] Pancheya T. V. Popoya L. P. Uzunoya A. N. (1996). Effects of salicylic acid on growth and photosynthesis in barley plants. J. Plant Physiol. 149, 57–63. doi: 10.1016/S0176-1617(96)80173-8

[ref37] Pasternak T. Groot E. P. Kazantsev F. V. Teale W. Omelyanchuk N. Kovrizhnykh V.. (2019). Salicylic acid affects root meristem patterning via auxin distribution in a concentration-dependent manner. Plant Physiol. 180, 1725–1739. doi: 10.1104/pp.19.00130, PMID: 31036755 PMC6752920

[ref38] Perrot-Rechenmann C. (2010). Cellular responses to auxin: division versus expansion. Cold Spring Harb. Perspect. Biol. 2:a001446. doi: 10.1101/cshperspect.a001446 20452959 PMC2857164

[ref39] Pokotylo I. Hodges M. Kravets V. Ruelland E. (2021). A ménage à trois: salicylic acid, growth inhibition, and immunity. Trends Plant Sci. doi: 10.1016/j.tplants.2021.11.008 [Epub ahead of Print]., PMID: 34872837

[ref40] Pokotylo I. Kravets V. Ruelland E. (2019). Salicylic acid binding proteins (SABPs): The hidden forefront of salicylic acid signalling. Int. J. Mol. Sci. 20:4377. doi: 10.3390/ijms20184377, PMID: 31489905 PMC6769663

[ref41] Rate D. Cuenca J. Bowman G. Guttman D. Greenberg J. (1999). The gain-of-function *Arabidopsis acd6* mutant reveals novel regulation and function of the salicylic acid signaling pathway in controlling cell death, defenses, and cell growth. Plant Cell 11, 1695–1708. doi: 10.1105/tpc.11.9.1695, PMID: 10488236 PMC144313

[ref42] Raz V. Koornneef M. (2001). Cell division activity during apical hook development. Plant Physiol. 125, 219–226. doi: 10.1104/pp.125.1.219, PMID: 11154331 PMC61004

[ref43] Rivas-San Vicente M. Plasencia J. (2011). Salicylic acid beyond defence: its role in plant growth and development. J. Exp. Bot. 62, 3321–3338. doi: 10.1093/jxb/err031, PMID: 21357767

[ref44] Rong D. Y. Luo N. Mollet J. C. Liu X. M. Yang Z. B. (2016). Salicylic acid regulates pollen tip growth through an NPR3/NPR4-independent pathway. Mol. Plant 9, 1478–1491. doi: 10.1016/j.molp.2016.07.010, PMID: 27575693 PMC7513929

[ref45] Santner A. Calderon-Villalobos L. I. Estelle M. (2009). Plant hormones are versatile chemical regulators of plant growth. Nat. Chem. Biol. 5, 301–307. doi: 10.1038/nchembio.165, PMID: 19377456

[ref46] Scott I. M. Clarke S. M. Wood J. E. Mur L. A. (2004). Salicylate accumulation inhibits growth at chilling temperature in *Arabidopsis*. Plant Physiol. 135, 1040–1049. doi: 10.1104/pp.104.041293, PMID: 15173571 PMC514138

[ref47] Seo D. H. Jeong H. Choi Y. D. Jang G. (2021). Auxin controls the division of root endodermal cells. Plant Physiol. 187, 1577–1586. doi: 10.1093/plphys/kiab341, PMID: 34618030 PMC8566267

[ref48] Serino L. Reimmann C. Baur H. Beyeler M. Visca P. Haas D. (1995). Structural genes for salicylate biosynthesis from chorismate in *Pseudomonas aeruginosa*. Mol. Gen. Genet. 249, 217–228. doi: 10.1007/bf00290369, PMID: 7500944

[ref49] Shakirova F. M. Sakhabutdinova A. R. Bezrukova M. V. Fatkhutdinova R. A. Fatkhutdinova D. R. (2003). Changes in the hormonal status of wheat seedlings induced by salicylic acid and salinity. Plant Sci. 164, 317–322. doi: 10.1016/S0168-9452(02)00415-6

[ref50] Silverman P. Seskar M. Kanter D. Schweizer P. Métraux J. P. Raskin I. (1995). Salicylic acid biosynthesis, conjugation, in rice' and possible role. Plant Physiol. 108, 633–639. doi: 10.1104/pp.108.2.633, PMID: 12228500 PMC157383

[ref51] Sprangers K. Thys S. Van Dusschoten D. Beemster G. T. S. (2020). Gibberellin enhances the anisotropyof cell expansion in the growth zone of the maize leaf. Front. Plant Sci. 11:1163. doi: 10.3389/fpls.2020.01163, PMID: 32849718 PMC7417610

[ref52] Tan S. T. Abas M. Verstraeten I. Glanc M. Molnár G. Hajný J.. (2020). Salicylic acid targets protein phosphatase 2A to attenuate growth in plants. Curr. Biol. 30, 381–395.e8. doi: 10.1016/j.cub.2019.11.058, PMID: 31956021 PMC6997888

[ref53] Tonnessen B. W. Manosalva P. Lang J. M. Baraoidan M. Bordeos A. Mauleon R.. (2015). Rice phenylalanine ammonia-lyase gene *OsPAL4* is associated with broad spectrum disease resistance. Plant Mol. Biol. 87, 273–286. doi: 10.1007/s11103-014-0275-9, PMID: 25515696

[ref54] Traw M. B. Bergelson J. (2003). Interactive effects of jasmonic acid, salicylic acid, and gibberellin on induction of trichomes in *Arabidopsis*. Plant Physiol. 133, 1367–1375. doi: 10.1104/pp.103.027086, PMID: 14551332 PMC281631

[ref55] Tsukaya H. (2019). Re-examination of the role of endoreduplication on cell-size control in leaves. J. Plant Res. 132, 571–580. doi: 10.1007/s10265-019-01125-7, PMID: 31321606 PMC6713683

[ref56] Van Butselaar T. Van den Ackerveken G. (2020). Salicylic acid steers the growth-immunity tradeoff. Trends Plant Sci. 25, 566–576. doi: 10.1016/j.tplants.2020.02.002, PMID: 32407696

[ref57] Vanacker H. Lu H. Rate D. N. Greenberg J. T. (2001). A role for salicylic acid and NPR1 in regulating cell growth in *Arabidopsis*. Plant J. 28, 209–216. doi: 10.1046/j.1365-313X.2001.01158.x, PMID: 11722764

[ref58] Vanstraelen M. Benková E. (2012). Hormonal interactions in the regulation of plant development. Annu. Rev. Cell Dev. Biol. 28, 463–487. doi: 10.1146/annurev-cellbio-101011-155741 22856461

[ref59] Vercruysse J. Baekelandt A. Gonzalez N. Inzé D. (2020). Molecular networks regulating cell division during *Arabidopsis* leaf growth. J. Exp. Bot. 71, 2365–2378. doi: 10.1093/jxb/erz522, PMID: 31748815 PMC7178401

[ref60] Vlot A. C. Dempsey D. A. Klessig D. F. (2009). Salicylic acid, a multifaceted hormone to combat disease. Annu. Rev. Phytopathol. 47, 177–206. doi: 10.1146/annurev.phyto.050908.135202, PMID: 19400653

[ref61] Wang Z. Rong D. Chen D. Xiao Y. Liu R. Wu S.. (2021). Salicylic acid promotes quiescent center cell division through ROS accumulation and down-regulation of PLT1, PLT2, and WOX5. J. Integr. Plant Biol. 63, 583–596. doi: 10.1111/jipb.13020, PMID: 33017089

[ref62] Weiss J. Gado-Benarroch L. Egea-Cortines M. (2005). Genetic control of floral size and proportions. Int. J. Dev. Biol. 49, 513–525. doi: 10.1387/ijdb.051998jw, PMID: 16096961

[ref63] Wildermuth M. C. Dewdney J. Wu G. Ausubel F. M. (2001). Isochorismate synthase is required to synthesize salicylic acid for plant defence. Nature 414, 562–565. doi: 10.1038/35107108, PMID: 11734859

[ref64] Wolters H. Jürgens G. (2009). Survival of the flexible: hormonal growth control and adaptation in plant development. Nat. Rev. Genet. 10, 305–317. doi: 10.1038/nrg2558, PMID: 19360022

[ref65] Wu Y. Zhang D. Chu J. Y. Boyle P. Wang Y. Brindle I. D.. (2012). The *Arabidopsis* NPR1 protein is a receptor for the plant defense hormone salicylic acid. Cell Rep. 1, 639–647. doi: 10.1016/j.celrep.2012.05.008, PMID: 22813739

[ref66] Xia J. C. Zhao H. Liu W. Z. Li L. G. He Y. K. (2009). Role of cytokinin and salicylic acid in plant growth at low temperatures. Plant Growth Regul. 57, 211–221. doi: 10.1007/s10725-008-9338-8

[ref67] Xie Z. Zhang Z. L. Hanzlik S. Cook E. Shen Q. J. (2007). Salicylic acid inhibits gibberellin-induced alpha-amylase expression and seed germination via a pathway involving an abscisic-acid-inducible *WRKY* gene. Plant Mol. Biol. 64, 293–303. doi: 10.1007/s11103-007-9152-0, PMID: 17390108

[ref68] Xu L. Zhao H. Y. Ruan W. Y. Deng M. J. Wang F. Peng J. R.. (2017). ABNORMAL INFLORESCENCE MERISTEM1 functions in salicylic acid biosynthesis to maintain proper reactive oxygen species levels for root MERISTEM activity in rice. Plant Cell 29, 560–574. doi: 10.1105/tpc.16.00665, PMID: 28298519 PMC5385951

[ref69] Zhang Y. L. Li X. (2019). Salicylic acid: biosynthesis, perception, and contributions to plant immunity. Curr. Opin. Plant Biol. 50, 29–36. doi: 10.1016/j.pbi.2019.02.004, PMID: 30901692

